# An SDN Focused Approach for Energy Aware Traffic Engineering in Data Centers

**DOI:** 10.3390/s19183980

**Published:** 2019-09-14

**Authors:** Paris Charalampou, Efstathios D. Sykas

**Affiliations:** School of Electrical & Computer Engineering, National Technical University of Athens, 9 Iroon Polytechniou Street, 15773 Athens, Greece

**Keywords:** software defined networking (SDN), data center, optimization, traffic engineering, energy awareness

## Abstract

There is a lot of effort to limit the impact of CO_2_ emissions from the information communication technologies (ICT) industry by reducing the energy consumption on all aspects of networking technologies. In a service provider network, data centers (DCs) are the major power consumer and considerable gains are expected by regulating the operation network devices. In this context, we developed a mixed integer programming (MIP) algorithm to optimize the power consumption of network devices via energy aware traffic engineering. We verified our approach by simulating DC network topologies and demonstrated that clear benefits can be achieved for various network sizes and traffic volumes. Our algorithm can be easily implemented as an application in the software-defined networking (SDN) paradigm, making quite feasible its deployment in a production environment.

## 1. Introduction

The problem of climate change due to global warming is already identified and the research community as well as industry are working on methods to limit its impact. The following areas have been identified as the main contributors of CO_2_ emissions: (a) energy production (29%), (b) transportation (27%), (c) industry (21%), (d) agriculture (9%) and (e) information communication technologies (ICT) (9%) [1]. In the area of ICT, a large increase that can reach a total of 15% is expected by the end of 2020 due to the deployment of 5G networks, mass introduction of IoT (Internet of Things) devices, IP traffic generated by video streaming and augmented reality applications. Data centers (DCs) in internet service providers (ISPs) account for more than 45% of the power consumption [2]. Although a marginal 10% of it is caused by network equipment [3], data center networking is responsible for 0.36% of the total power consumption [4]. This figure is expected to rise even more because of NFV (Network Functions Virtualization) and software defined networking (SDN) based service deployment [5]. In 5G networks, the number of DCs is expected to rise in order to support mobile edge computing (MEC) and user plane intensive applications [6]. Also, the majority of legacy applications will be migrated to cloud infrastructure increasing the workloads served by DCs.

Data center networks are characterized by traffic patterns and volumes that significantly vary from typical IP based networks that reside in ISP’s premises. Typically, DC networks are overprovisioned with a large number or redundant devices and links. Depending on the deployed topology, access and aggregation layer links rarely exceed 10% of utilization due to the high number of redundant links on these layers [7]. Links and devices on higher layers of the topology (closer to root or data center routers) tend to have higher levels of utilization and thus consume more energy. Accordingly, only 60% of installed links could potentially serve traffic even during a busy hour [8]. 

If we consider the utilization of available resources during a whole day and not only during the busy hour, DC network infrastructure stays in idle state serving zero to minimal traffic (mostly for management purposes and maintaining the routing algorithms) for almost 70% of the day. Even on higher traffic volumes, the average link utilization is not surpassed for the remaining 25% of the day. Since DC networks are mainly constructed to provide a high level of resilience during a busy hour, we identify the opportunity to minimize the power consumption of network devices and links between them for the largest part of the day.

In this paper we address the problem of minimizing the power consumption within DCs. The main obstacle in developing a practical optimization method for DCs is the requirement for global knowledge of network topology and the flows matrix between the hosts. DC networking presents high variability mostly from virtual machines (VMs) instantiation that have ephemeral life span in cloud environments. Thus, there is a need to continually monitor for new traffic flows and the optimization algorithm should act accordingly. Building a distributed algorithm for this purpose is not appropriate since it would require the introduction of new or updated protocols. Nevertheless, SDN architectures are already deployed in production environments and the centralized implementation of SDN controllers (SDN-C) now allows practical implementations of such optimization.

Taking for granted that all modern DCs follow the SDN architecture, we show that, via energy aware traffic engineering, an SDN [9] application can successfully address the problem. Depending on hardware properties and traffic conditions, links can be put on a lower power state or completely shut off via traffic steering. We formulate the optimization process as a mixed integer programming (MIP) problem [10] that models power consumption taking into account all relevant constraints. Using standard optimization tools and heuristics for its solution we show that significant power savings result. Since the solution is optimal only for a short time frame, as long as traffic loads do not change considerably, periodical repetitions and fast execution times are required and fine tuning of the solver is mandatory. We took great care to ensure that the optimization algorithm can be executed in a timely manner and confirmed the viability of our approach on a variety of topologies and network sizes. Finally, we implemented an application following the SDN paradigm to demonstrate the applicability of our approach and verified it against emulated topologies for performance evaluation and experimental ones for software verification purposes. 

## 2. Related Work

For the ICT domain, the main method to limit CO_2_ emissions is by decreasing the power consumption of network topologies. A lot of work is performed in this area on various layers of the network architecture. Existing methods can be classified into two main categories (a) evolving hardware to support power consumption proportional to traffic served [11] and (b) putting unused links and devices into sleep mode after applying traffic engineering techniques to reroute traffic [12].

Techniques to allow unused links to get into sleep states can be applied to ‘access’ part of the network (both wired and wireless), to ‘transport’ (covering wired aggregation and mobile backhauling based on optical links) and to ‘core’ [13]. Based on work performed in core networks, a number of mechanisms have been proposed and that can be separated into four main categories (a) new routing protocols [14], (b) traffic engineering techniques [15], (c) new network architectures [16], and (d) modifications to existing routing protocols [17,18] so that routing decisions will be based on energy consumption criteria. The main drawback of existing approaches is the requirement for full knowledge of network topology and link utilizations. Even though getting a full layer-3 topology is rather easy even on large scales networks, it is not feasible to gather per flow statistics even when utilizing modern monitoring systems with telemetry [19] and big data analytics [20].

On the DC domain, a more holistic approach is taken into consideration that addresses the power consumption not only on networking and computing devices but on supporting equipment such as cooling devices. On the networking layer, similar approaches have been proposed that try to put unused links in sleep state. Furthermore, they only focus on legacy devices with sleep states, neglecting other possible power states [21]. In contrast, modern switches and especially fiber optics links in DCs support a certain degree of energy proportionality with a step-wise approach [22]. A typical modular network device consumes power only in two concrete states (a) maximum power when operating in normal mode and (b) minimum power when operating in sleep state. This mode of operation is applicable on chassis layer, line cards and routing processors. There is no direct correlation between traffic volumes and power consumption, but only on environmental aspects like DC room temperature and the number of routing table entries. Interfaces on the other hand operate on energy aware states according to the amount of traffic they serve. The number of states and the maximum power consumption of an interface varies among manufacturers and depends on the link type (multimode optics, single mode optics, DAC cables, CAT-6 copper cables) [23].

Until recently, all proposed mechanisms have been applied only to experimental topologies and lack adoption from major networking equipment manufacturers. In addition, there is a lack in real life deployments of such solutions due to a number of reasons: (a) protocol expansions are not eagerly adopted from manufactures, (b) network statistics (on per flow basis) and topology monitoring cannot scale well, and (c) advances in the area of SDN are not taken into account.

The SDN architecture [24] of separated data and control plane has been successfully used to build networks inside a DC regardless of the purpose served and the workloads hosted, from 5G and NFV services to generic IT applications and data storage. Furthermore, SDN already provides the mechanisms to efficiently collect measurements and apply forwarding rules to existing equipment without modifications to devices or routing protocols. Finally, SDN design allows to easily integrate external applications via well-defined software APIs in SDN controllers (SDN-C). Therefore, the use of an SND-C application seems to be the only viable method for network policies in any aspect of DC networking.

In this paper we pursue the SDN paradigm as applied for traffic engineering purposes, so as to mitigate the aforementioned gaps. First, we define an optimization problem that takes into account new hardware capabilities by modeling the interface power consumption in concrete power states. Then we show that the optimization problem can be solved in a timely manner. Finally, by harvesting SDN capabilities we get the opportunity to develop an optimization application using a real, production ready SDN-C and to apply the optimal solution in a scalable and practical manner. Our optimal solution can be applied without traffic loss and is independent of the traffic patterns (UDP or TCP based).

Relevant approaches [8] consider only selected topologies, are optimized for these and assume that there is a-priori knowledge of traffic patterns. Even though they make use of SDN as a method to apply the optimal solution, they do not fully exploit SDN capabilities. In particular, they resort on legacy methods to collect statistics via SNMP. As a result, they cannot lead to practical implementations in production environments. Finally, since their solution is developed as a part of the SDN-C, not as an application following SDN paradigm, it is coupled only to the specific implementation and cannot be scaled to other controllers and topologies.

## 3. Optimization Problem

We formulate the following optimization problem to determine the optimal topology and state of links (either disabled or in an optimal state for served traffic). We consider only fixed sized network devices since this is the trend in currently deployed DC network topologies. Furthermore, since we envisage our approach to be deployed in real-life scenarios, we exclude the option to put into sleep mode a whole device.

Definitions:

*V*: set of network devices in a given topology. Device υ∈V has a base power consumption of B_υ_

*E*: set of links between the devices. Links are assumed to be bi-directional thus link *l* = $\left(\upsilon ,\upsilon^{\prime }\right)$ originates from device υ and terminates at device υ’ where υ,υ′∈V

*F*: set of flows from Access only devices. Flow (s,t)∈F originates from device *s* and terminates at device *t* where s,t∈V

*ST*: set of possible power states *n* for a link

$P_{l}^{\left(n\right)}$: power consumption of link *l* when operating in state *n*

$C_{l}^{s}$: capacity of link l∈E operating on a state n∈ST

$x_{l}^{\left(s,t\right)}$: flow from *s* to *t*, where s,t∈V, passing through link l∈E

$\Phi_{l}\left(r\right)$: power consumed at link l∈E when carrying traffic *r*

$\tau_{\left(s,t\right)}$: flow from device *s* to *t* where s,t∈V

Decision Variables:

$S_{l}^{\left(n\right)}$: a binary variable vector to describe the state of a link. Value $S_{l}^{\left(n\right)}$ equals to 1 if link *l* is in state *n*

$y_{v}$: a binary variable describing the state of device *υ* and equals to 0 when a device is in sleep state

$z_{l}$: a binary variable defining if link *l* is in sleep mode and equals to 1 when is serving any volume of traffic

Our objective is to minimize the following gain function that calculates the total power consumed from network devices

(1) $$\mathrm{Minimize}:\;\sum_{v\in V}B_{v}\cdot y_{v}+\sum_{l\in E}\sum_{n\in ST}\;S_{l}^{\left(n\right)}\cdot P_{l}^{\left(n\right)}$$

While maintaining the following constraints:

(2) $$\underset{\left(\upsilon ,\;\upsilon^{\prime }\right)\in E}{\sum }x_{\left(\upsilon ,\upsilon^{\prime }\right)}^{\left(s,t\right)}-\underset{\left(\upsilon ,\;\upsilon^{\prime }\right)\in E}{\sum }x_{\left(\upsilon \prime ,\upsilon \right)}^{\left(s,t\right)}=\{\begin{array}{ll} \tau_{s,t} & if\;\upsilon =s\\ -\tau_{s,t} & if\;\upsilon =t\\ 0 & otherwise \end{array},\forall \;\left(s,t\right)\in F\;and\;\upsilon \in V$$

(3) $$\underset{\left(s,\;t\right)\in F}{\sum }x_{l}^{\left(s,t\right)}\leq C_{l}^{\left(n\right)},\;\forall \;l\in E,\;n\in \mathrm{ST}$$

(4) $$z_{l}\cdot C_{l}^{\left(n\right)}\geq \underset{\left(s,t\right)\in F}{\sum }x_{l}^{\left(s,t\right)},\;\forall \;l\in E,\;n\in \mathrm{ST}$$

(5) $$z_{\left(\upsilon^{\prime },\;\upsilon \right)}=z_{\left(\upsilon^{\prime },\;\upsilon \right)},\forall \;l=\left(\upsilon ,\upsilon^{\prime }\right)\in E$$

(6) $$y_{\upsilon }\cdot \left(\sum_{\left(\upsilon ,\upsilon^{\prime }\right)\in E}C_{\left(\upsilon ,\upsilon^{\prime }\right)}^{\left(n\right)}+\sum_{\left(\upsilon^{\prime },\upsilon \right)\in E}C_{\left(\upsilon^{\prime },\upsilon \right)}^{\left(n\right)}\right)\geq \sum_{\left(s,t\right)\in F}\left(\underset{\left(\upsilon ,\upsilon^{\prime }\right)\in E}{\sum }x_{\left(\upsilon ,\upsilon^{\prime }\right)}^{\left(s,t\right)}+\sum_{\left(\upsilon \prime ,\upsilon \right)\in E}x_{\left(\upsilon^{\prime },\upsilon \right)}^{\left(s,t\right)}\right)$$

(7) $$C_{l}^{\left(n-1\right)}\leq \underset{\left(s,t\right)\in F}{\sum }x_{l}^{\left(s,t\right)}\leq C_{l}^{\left(n\right)}$$

(8) $$\underset{l\in E,n\in ST}{\sum }S_{l}^{\left(n\right)}=1$$

(9) $$\Phi_{l}\left(r\right)=\underset{n\in ST}{\sum }S_{l}^{\left(n\right)}\cdot P_{l}^{\left(n\right)}$$

Equation (2) guarantees flow preservation, i.e., flow that enters a device is also coming out of it without packet loss. Equation (3) guarantees that capacity constrains are not violated and the sum of all the flows coming from an interface cannot be greater than the capacity of the interface. The fact that a link must be on a specific state and not on sleep mode and in parallel be on the same state on both interconnected devices is defined in Equations (4) and (5). Equation (6) guarantees that a device (and the interconnected one) are kept online if they serve any amount of traffic. Capacity constraint in Equation (7) ensures that the traffic through an interface is not grater then the capacity for a given state. Each interface can only be on one state and this is guaranteed by Equation (8). Equation (9) expresses the consumption of a link based on the power state defined by the traffic crossing each interface.

This is a typical MIP (mixed integer programming) problem and the solution is relatively fast in case of small topologies with a limited number of devices and interconnecting links. Network topologies in DCs are much larger with a high number of nodes (hundreds of switches and thousands of links) and solving the above optimization problem becomes time consuming. Thus, CPLEX solver [25], a well-known solver for optimization problems and constrained programming for linear and integer programming problems, was used to apply a number of heuristics and produce a suboptimal solution in a timely manner. In addition, flows and capacity values are expressed as integers (bytes per second) to accelerate CPLEX execution times.

CPLEX MIP solver applies a number of algorithms to automatically select the best method to solve complex problems. Our problem is by definition feasible since traffic loads can be served in the initial network state. That is, if links are operated on higher energy state, all constrains are fulfilled. In our work, CPLEX as MIP solver uses pre-processing and probing by setting all binary variables to either 0 or 1 and checking the logical implications. It automatically selects an appropriate Branch and Cut algorithm to solve the optimization problem. If heuristics are required, a neighborhood exploration search starts which is called solution polishing after the time limit is reached. Polishing is based on the integration of an evolutionary algorithm within an MIP branch and bound framework.

Since we aim at practical implementations, we applied a hard time limit to the execution of the algorithm. A number of parameters were evaluated in order to get a solution in a timely manner. Initially we obtained a sub-optimal solution (that reduces the total power consumption) and then explored for a better one without violating the timing restriction. The CPLEX parameters evaluated are shown in the following Table 1.

The first parameters were selected so as to fit the available hardware resources where CPLEX was executing. In more detail, *threads* value was set to 8 to fully utilize available vCPU capacity of hosting VM. *Tilim* was set to a value that can produce a viable solution even on large data set. *Mipephasis* was set to feasibility mode in order to produce an initial suboptimal value given the timeframe and later search for the optimal value. *Probe* was set to the highest possible value since the initial time spend looking for a suboptimal solution guaranteed that we could have power savings for all scenarios. The other parameter (*varsel*, *lbheur* and *fpheur*) values were selected to fit the nature of the problem and the available input data. Multiple executions of the algorithm on the same data set were performed to find an optimal set considering the time limitations imposed in a production grade network environment.

## 4. Emulation Results

We evaluated the results of our optimization algorithm on various network topologies, sizes (number of nodes) and traffic volumes. We emulated three different network topologies applicable to DC networking (a) classical three tier topology [26], (b) fat tree [27], and (c) leaf and spine [28]. We focused only to inter-switch communications and did not consider the impact of multihomed servers where further benefits can be achieved. Physical topologies and switches were emulated using Mininet [29] that implements OpenvSwitch (OVS) [30] as an Openflow switch, because of its ability to create and maintain large network topologies [31]. Mininet is responsible for instantiating a number of Openflow enabled switches, connecting them to an external SDN-C, creating a number of physical hosts and interconnecting them with virtual links. Mininet allows the development of scripts to deploy large scale topologies based on pre-defined parameters. In this context, we developed a series of scripts that automatically generate the topologies under examination with the number of devices described in Table 2.

**Figure 1:**
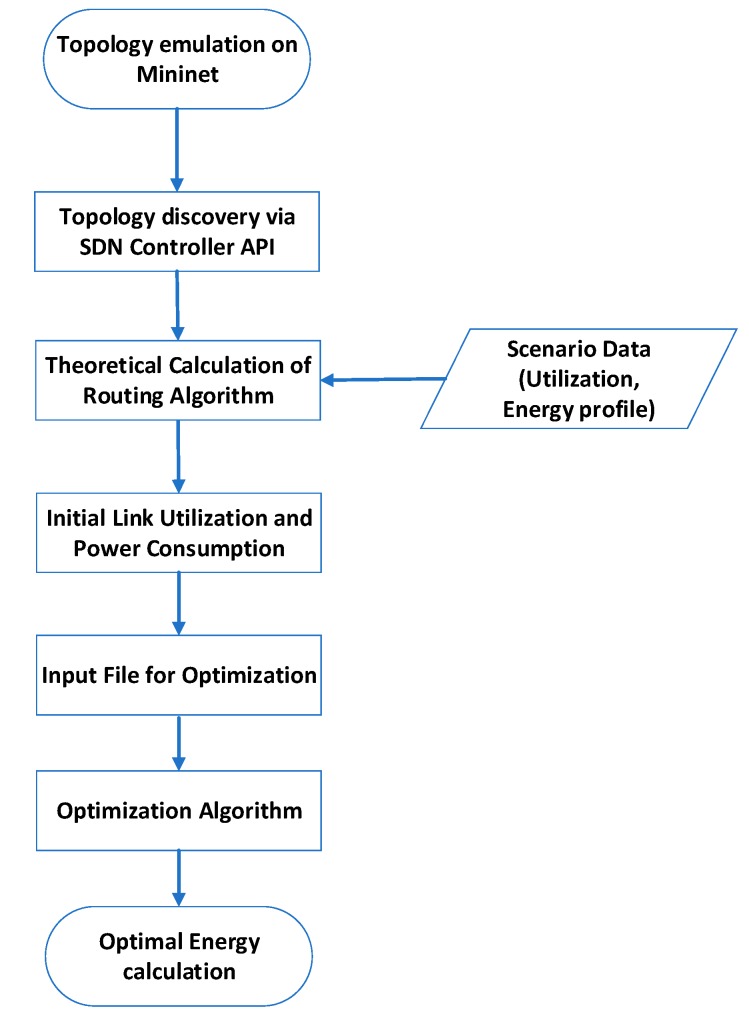
Process followed during the simulation.

We have chosen Opendaylight (ODL) [32] as the SDN controller and we executed our experiments employing ODL’s RESTful API. The experimentation procedure is depicted in Figure 1. Initially we spin up the desired topology on a VM instance executing Mininet software. The OVS instance inside Mininet is connected to an external VM that runs ODL. A python module collects the topology information and the configured link capacities. The CPLEX solver runs on a separate dedicated VM in order not to interfere with ODL. Our software module is fed with the device power consumption model and randomized flows (depending on the scenario) so as to calculate the initial power consumption and link utilization according to current, non-optimized state where all links operate on the highest possible state. The same module includes the routing functionality and generates traffic flows between all devices. Based on the scenario, the appropriate input configuration file for CPLEX is generated and the MIP solver is invoked. According to the results, new optimal consumption and link utilizations are calculated, and the new topology is stored in an external file for review.

The power consumption model of an interface consists of a baseline value due to the transceiver and a traffic proportional part due to the electronics parts on the Linecards. Typical values for transceiver consumption start from 1.5 W per transceiver for a 10 Gbps multimode optical module till 3.5 W/module for 100 Gbps single mode fiber [33]. On the other hand, the nominal value on the switch side is 3.5 W for a 10 Gbps interface and 14 W for a 40 Gbps one. In our emulation, a non-linear power model is assumed where the initial states tend to consume higher volumes of power. We considered four different states regarding the link power consumption, where the last state corresponds to the maximum power consumption of a link. The link is considered to consume no power when in sleep state. The first step includes the power requirements to maintain the link state (thus consumes relatively more than intermediate steps). The next steps are according to power consumption data sheet from switch manufacturers [34].

Network sizes varied from 12 switches (4 server racks equivalent in real deployments) to 74 switches (32 server racks) were emulated. For each topology the exact figures for switches per network layer are listed in Table 2. Traffic profile (we evaluated only east–west communications) and total traffic volume were considered constant for each execution of the experiment.

**Figure 2:**
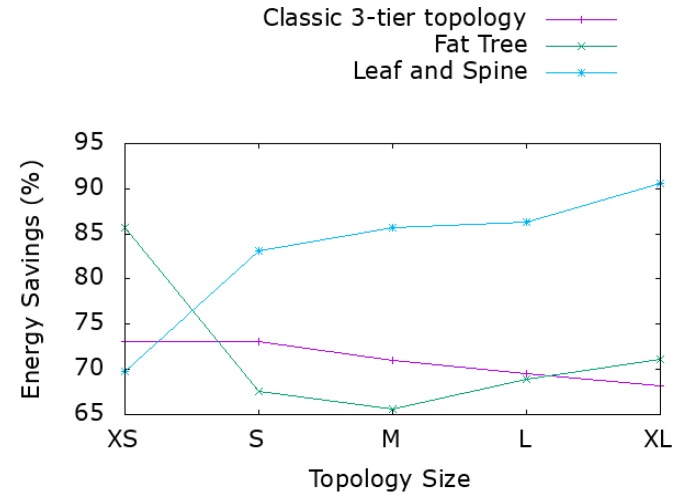
Energy savings for various network topologies and sizes.

Figure 2 demonstrates the results for various network sizes and different network topologies. Even though there is a different level of redundancy, starting from limited resiliency in a 3-tier architecture up to the highest level of resiliency in leaf and spine topology, we recorded a power reduction of at least 65% in the worst case. By adding redundant paths, especially on the spine layer, the power savings increased up to 90%. Leaf and spine topology by design includes the higher number of redundant links compared to the other two topologies. Adding switches on the spine layer increases the number of redundant paths without serving more hosts and thus increases the power savings potential. There is no similar improvement in the other topologies although savings are around 70%. The classic 3-tier architecture has less redundant links. Energy savings derive from partially loaded link power states and are independent of the number of switches in each layer. In the fat tree architecture, where the number of alternate paths depends on the switches at the aggregation level, power consumption is affected only by the number of devices at this layer.

**Figure 3:**
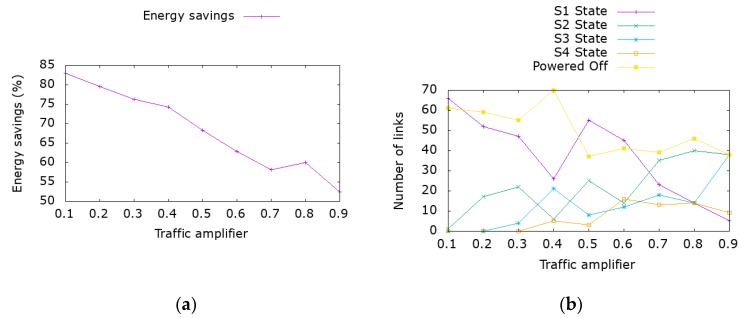
Analysis for average size leaf and spine topology under various traffic loads. (**a**) Energy savings from optimization algorithm. (**b**) Number of interfaces per state after the execution of the algorithm.

On the leaf and spine topology, the most promising in terms of power savings potential, we examined the impact of various workloads. To compare different traffic volumes we introduce the concept of traffic amplifier, being a percentage of the same maximum traffic across all topologies. The maximum traffic is calculated assuming that hosts load the network as much as possible without violating the link capacity on a given topology. Traffic amplifier is the factor used to multiply the reference flows between the hosts (the ones resulting in full core link utilization without traffic engineering algorithm in place). Thus, a traffic factor of 10% corresponds to a random generation of flows between the hosts that lead to a maximum 10% utilization on the core links. Figure 3 depicts the impact of increasing traffic volumes on the energy savings in conjunction with the number of interfaces that operate on specific power states. The power state is derived from the traffic level at each interface, whereas on the 1st state the load is 25% of the nominal and the 4th state corresponds to the maximum capacity.

As depicted in Figure 3, even for high load utilization, where a number of links have to operate on their maximum capacity, we still recorded energy savings up to 52%. Notice that, since the maximum gains are achieved when a link is at sleep state, the MIP solver tends to reroute traffic on selected links causing them to operate at higher rate, instead of distributing the load to multiple links which is the current mode of operation in existing DC deployments. As traffic increases, the margins for energy savings via traffic engineering are narrower as shown in Figure 3a where energy savings decrease as link utilization increases from 85% savings to nearly 50%.

**Figure 4:**
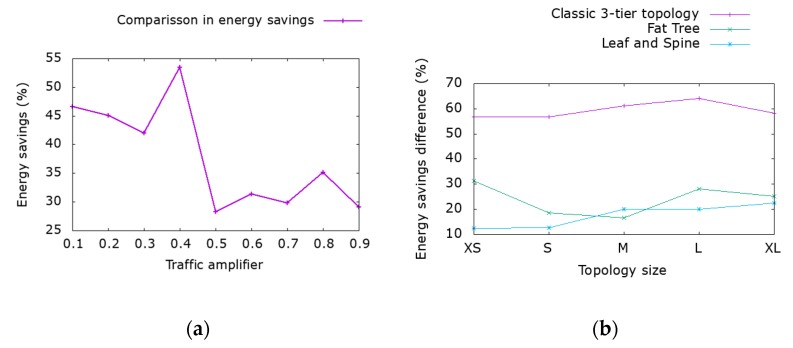
Comparison between approaches based on disabling interfaces and our interface power scale proposal. Difference under (**a**) various workloads on leaf and spine topology, and (**b**) various topologies and number of devices for the same workload.

Next, we evaluate the gains from considering enhanced power scaling capabilities of interfaces compared to only disabling unused interfaces and devices. In Figure 4a, we plot the energy savings gain versus traffic volume achieved in a leaf and spine topology. Even for high traffic volumes, there is at least 28% benefit when taking into account the interface’s power state behavior. Similar results can be achieved regardless of the selected topology as demonstrated in Figure 4b. The benefits of interface power scaling decrease as traffic increases since more interfaces operate closer to their maximum capacity consuming the maximum power. Power scaling is far more beneficial in the classic 3-tier topology due to the absence of redundant paths on core links and the fact that only a small portion of the links can be disabled. In the fat tree and leaf and spine topology, benefits are still significant compared to the benefits of disabling only unused interfaces even though a large number of redundant links can be switched off. Figure 4 demonstrates that a combined strategy of traffic engineering (to disable the highest number of interfaces) and exploiting interface power states is required so as to achieve the maximum benefits in power savings since greater values of power savings can be achieved regardless of the traffic volumes and topology size.

We further compare our results to existing studies that do not consider hardware capabilities and only try to suspend unused interfaces. We selected the studies based on the relevance to our study and the methodology followed. Existing literature in this domain can be classified into two main categories: (a) studies that can be applied to all types of networks [35], (b) studies that focus only on DC topologies. In both cases full knowledge of the topology and the relevant traffic matrix is required. The optimal solution is applied either via SDN methods or following legacy approaches, i.e., by modifying the routing information. Since there is no direct comparison on topology level with the studies in the first category, we can only examine power savings achieved for equivalent average link utilizations of 10%, 50% and 90%. Compared to the results in Figure 6 of [35], our method can provide five times more energy savings in the worst-case scenario of 90% link utilization, achieving 53% power savings instead of only 10%. Considering 50% link utilization, we achieve more than triple savings namely 68% in our case compared to 22%. For low link utilizations of 10% the savings we get are more than double, 85% savings in our study compared to 35%.

Compared to studies in the second category, we can consider the same traffic volumes and network topology. In particular, we compared our method towards the fat-tree network topology examined in [8] under mid-traffic profile (50% on near nodes, 50% on far nodes) for 1024 nodes and maximum link utilization of 20%. In this case, our approach can produce around 45% more savings, i.e., 65% instead of 45% power savings. For lower number of hosts and similar link utilization, we observe 55% more savings when our approach is applied. Based on the aforementioned comparison with DC focused methods, the energy savings potential of our method is far greater compared to approaches that try to optimize power consumption in DCs without considering hardware characteristics.

## 5. SDN Application

In SDN architecture, control and forwarding planes are clearly separated defining a discrete device for control plane functions, the SDN controller (SDN-C), and keeping the forwarding process on the physical switches. This architecture, as defined by ONF (open networking foundation), allows the development of SDN applications that harvest controller APIs to collect statistics or routing information, to modify port configuration and to reroute traffic. To demonstrate the applicability of our approach in a DC environment, we developed an application that can be easily integrated to any SDN controller.

**Figure 5:**
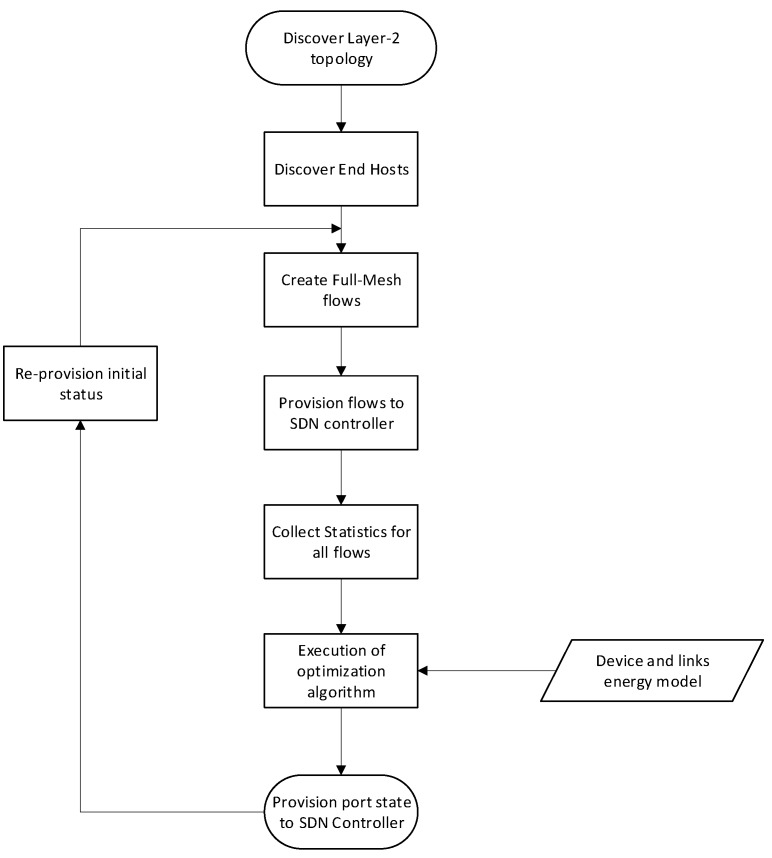
Software defined networking (SDN) application internal sequence of activities.

The flow chart in Figure 5 depicts a high-level description of the internal activity of such an SDN application. First, it discovers all relevant switches and hosts that reside in the SDN-C database. Then, a full mesh list of flows between hosts is generated for the given topology and their values are stored internally in application’s configuration. In accordance with the Layer-2 topology as created by the SDN-C using standard STP (Spanning Tree Protocol) algorithms, a set of link flows results. The SDN application provisions these flows without modifying the existing routing information. On a configurable time interval, the application collects flow statistics with the Openflow build-in mechanism. Based on flow statistics and power profile for each device type (power consumption per state), the CPLEX module calculates the new optimal state for all device and interfaces. The new topology is then provisioned to devices directly or via the SDN-C. After a sleeping period, the whole process is executed again removing all host specific flows generated on the initial execution. All ports and devices are re-provisioned on the initial state where they consume the maximum amount of energy and can serve nominal traffic.

Note that redundant links remain in service only if there is enough traffic in the topology. In general, as expected from similar approaches in core networks, a certain stretch on the path length between end devices is expected accompanied by a minor degradation in service quality. Since communication inside DCs involves a small number of hops, especially in leaf-and-spine topology, this stretch is not expected to affect applications. The SDN application guarantees that connectivity of end hosts is not disrupted at any case. 

The key point is that the optimization problem can be solved by an external system irrespective of the software architecture and programming language of the controller. Nevertheless, a number of additional software components are needed. In order to verify our proposal, an SDN application coded in python, was deployed at NTUA’s Computer Networks Laboratory using a testbed consting of Opendaylight version 7.3 SDN controller, an HPE switch running firmware WB.16.05.0003 and several software implementations based on OpenVSwitch. In particular, we have developed the following generic modules:

Topology_discovery: This module uses the RestAPI of an SDN-C to automatically discover all openflow enabled switches, interconnection links between the devices and end-hosts for any topology. The outcome of the discovery process is stored in a single file, The SDN-C is scanned periodically for topology changes.

Flow_generator: Based on the topology and hosts discovered, the flow_generator module creates a full table of traffic flows between end hosts. Communication between Openflow [36] switches is omitted as it is expected to consist of management traffic, marginal compared to the volume of production traffic. These flows are provisioned via Rest API of the SDN-C to all devices.

Stats_Collector: It runs periodically to collect the statistics and aggregates the results according to the operator needs. Stats_Collector uses the build-in mechanism of Openflow protocol and gathers the values of flow counters based on “Counters” field for the provisioned flows of the previous step. 

Green_Topology_Optimizer: This is the CPLEX module performing the optimization and some python modules to control its execution. Based on a preconfigured link power consumption model and the configuration files as created from Topology_discovery and Stats_collector modules, this module generates the optimal power state for all devices and interfaces.

Port_Modifier: According to the solution generated, this module provisions the new state either via the SND-C programmable API, via OVSDB or via ovs-vsctl.

**Figure 6:**
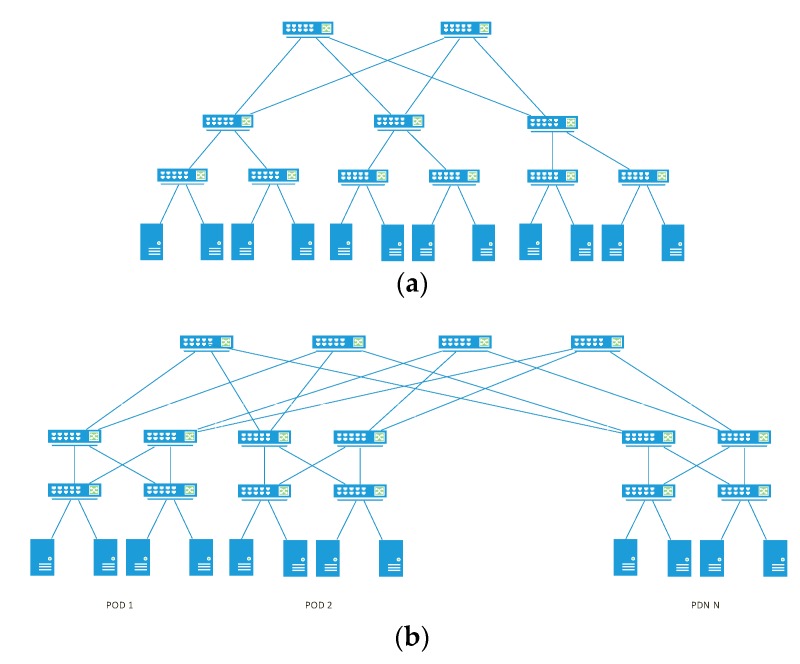

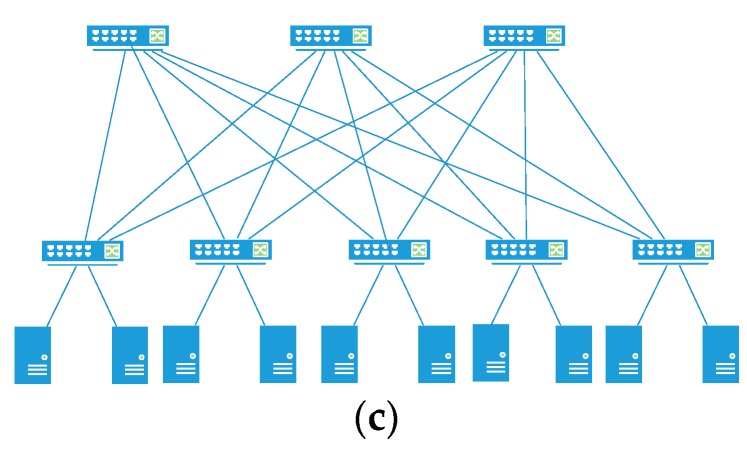
Network topologies under evaluation: (**a**) classical-3-layer (**b**) fat tree (**c**) leaf and spin.

The verification of our application was performed into two steps. The first step, the functional verification, consisted of the successful integration of the above compents in the physical and virtual lab environment. The second step, the performance evaluation, was carried out on the same Mininet emulated topologies as in Section 4. Thus, the optimal solution in terms of power consumption savings for each topology does not change. Topologies emulated by Mininet are depicted in Figure 6.

The network size of emulated topologies affects the time required for our SDN application to calculate and provision the optimal solution. Thus, we evaluated its total execution time and not only the optimization (CPLEX) part. We performed measurements for the concrete phases: (a) time required to discover network topology and compute link power consumption based on the device’s energy model and link capacity; (b) flow generation for full mesh communication among hosts and their provisioning via Openflow commands; and (c) collection of flow statistics and initialization of optimization problem CPLEX solution. As shown in Table 3, the topology discovery part is the faster phase even for large topologies. The times for the flow generation and the collection of flow statistics depend only on the number of flows and end hosts and are independent of the complexity of the topology. The two initial phases have to be executed sequentially in less than 100 s in the worst case scenario. After the bootstrap, the statistics collection can forked to different processes. Measured values appearing in table must be regarded as the upper limit for this phase.

Timely execution of the SDN application is of high importance since we aim at a practical implementation. As demonstrated in Table 3, execution is fast even when the MIP optimization is applied to large network topologies. Next, we compare the performance of our solution to a similar approach in existing literature [37] that requires full knowledge of the flow matrix and applies heuristic algorithms (referred to as QRTP and RQRTP) for traffic engineering. The method in [37] is selected for comparison since it is developed as an SDN application like ours, it is focused on DC network topologies and requires equivalent input for the optimization problem (flow matrix). Applying traffic engineering decisions is based on QoS and network performance metrics, not on power saving criteria. Since the comparison cannot be direct for the full scope (energy savings and complexity) of our study, we compare only the execution times of the optimization algorithm, i.e., the time to calculate the optimal solution for the same number of flows and the same network topology. Our optimization algorithm can be solved significantly faster for small number of flows. Namely, for 100 flows, we need 0.08 s to calculate the optimal solution in our algorithm compared to 3.16 and 2.66 s, respectively, for the QRTP and RQRTP algorithms, using equivalent hardware resources (i.e., number of CPUs) while emulating a classical 3-layer network topology. The number of hosts and traffic volumes do not impact the complexity of the algorithms thus are not mentioned in detail. For larger topologies and 500 flows, our solution generates an optimal solution in 1.57 s whereas QRTP in 178.13 and RQRTP in 41.45 s. In the extreme scenario of 1000 flows, our optimization problem can be solved in 3.08 s compared to 1227.26 and 46.07 s respectively, for QRTP and RQRTP, and the same type of topology.

## 6. Conclusions

Due to the nature of DC network topologies, deployments tend to be overprovisioned with sparse utilization even in peak hour. We showed that DC networking can be largely optimized regarding power consumption regardless of the topology selected. The benefits in power consumption range from 65% to 90% in all typical scenarios depending on the total load. Since power benefits are coupled with traffic volumes, harvesting hardware capabilities for traffic steering can guarantee these savings even for high workloads reaching 50% for fully utilized leaf and spine topology which is the benchmark topology for DCs. Furthermore, we demonstrated that our proposal is a viable solution for DCs where SDN is deployed. It can be implemented as an SDN application regardless of network equipment manufacturer and SDN controller user and therefore easily applied to real life deployments.

## Figures and Tables

**Table 1:** Description of CPLEX software parameters that were considered during the execution of optimization algorithm.

Parameter	Description
tilim	Duration in seconds that CPLEX looks for a solution to the optimization problem
threads	Manages the number of parallel threads used during the calculations (maximum value depends on the available CPUs)
parallelmode	Sets the parallel optimization mode. Possible modes are automatic, deterministic, and opportunistic
mipemphasis	Controls trade-offs between speed, feasibility, optimality, and moving bounds in MIP.
probe	Sets the amount of probing on variables to be performed before MIP branching. Higher settings perform more probing
varsel	Sets the rule for selecting the branching variable at the node which has been selected for branching
lbheur	Controls whether CPLEX applies a local branching heuristic to try to improve new incumbents found during a MIP search
fpheur	Turns on or off the feasibility pump heuristic for mixed integer programming (MIP) models

**Table 2:** Number of devices on evaluated scenarios.

Size	Access (Number of Switches)	Aggregation (Number of Switches)	Core (Number of Switches)
**Classical 3-Layer**			
Size 1 (XS)	8	2	2
Size 2 (S)	16	4	2
Size 3 (M)	32	4	2
Size 4 (L)	64	4	2
Size 5 (XL)	64	8	2
**Fat Tree**			
Size 1 (XS)	8	2	2
Size 2 (S)	16	4	2
Size 3 (M)	32	4	2
Size 4 (L)	64	4	2
Size 5 (XL)	64	8	2
**Leaf and Spine**			
Size 1 (XS)	8	2	n/a
Size 2 (S)	16	4	n/a
Size 3 (M)	32	4	n/a
Size 4 (L)	32	8	n/a
Size 5 (XL)	64	8	n/a

**Table 3:** Performance evaluation of SDN application.

Size	Topology Discovery (s)	Flow Provisioning (s)	Statistics Collection (s)
**Classical 3-layer**			
Size 1 (XS)	0.45	0.942	0.808
Size 2 (S)	0.825	3.649	3.313
Size 3 (M)	1.495	17.059	13.203
Size 4 (L)	2.495	76.116	71.189
Size 5 (XL)	3.739	71.941	72.298
**Fat Tree**			
Size 1 (XS)	0.608	0.995	0.879
Size 2 (S)	1.198	3.916	3.258
Size 3 (M)	5.068	35.099	23.061
Size 4 (L)	5.024	89.483	72.062
Size 5 (XL)	4.539	79.729	70.433
**Leaf and Spine**			
Size 1 (XS)	0.599	0.952	0.856
Size 2 (S)	1.72	4.316	3.277
Size 3 (M)	3.342	17.562	13.587
Size 4 (L)	6.5	18.853	13.815
Size 5 (XL)	13.556	78.15	71.851
